# Inhibitory activities of acteoside, isoacteoside, and its structural constituents against protein glycation in vitro

**DOI:** 10.1186/1999-3110-54-6

**Published:** 2013-08-19

**Authors:** Yuh-Hwa Liu, Yeh-Lin Lu, Chuan-Hsiao Han, Wen-Chi Hou

**Affiliations:** 1 Division of Gastroenterology, Shin Kong Wu Ho-Su Memorial Hospital, Taipei, Taiwan; 2 Department of Primary Care Medicine, Taipei Medical University, Taipei, Taiwan; 3 School of Pharmacy, Taipei Medical University, Taipei, Taiwan; 4 Traditional Herbal Medicine Research Center, Taipei Medical University Hospital, Taipei, Taiwan; 5 Graduate Institute of Pharmacognosy, Taipei Medical University, Taipei, Taiwan

**Keywords:** Acteoside, Advanced glycation endproducts, Argpyrimidine, Cafeic acid, N^ϵ^-(carboxymethyl)lysine, 3′,4′-dihydroxyphenylethanol, Isoacteoside, Methylglyoxal

## Abstract

**Background:**

Advanced glycation end products (AGE) are substances that can induce insulin resistance in adipocyte, hepatocyte and muscle cells. This resistance correlates highly with cardiovascular disease and diabetic complications. Acteoside (A), a phenylethanoid glycoside, is an active compound in several plants and traditional herbal medicines. Acteoside, its structural isomer, isoacteoside (I), and their constituents, caffeic acid (C) and 3,4-dihydroxyphenylethanol (D), were used in the study to investigate the inhibitory activity against AGE formations *in vitro*.

**Results:**

AGE formations were detected by anti-(N^ϵ^-(carboxymethyl)lysine (anti-CML), using bovine serum albumin (BSA)/glucose (glc) and BSA/galactose (gal) as models, or by anti-argpyrimidine (anti-AP), using BSA/methylglyoxal (MGO) as models. It was found that A, I, C, or D, each at 5 mM, could attenuate the CML formations detected by ELISA in the BSA/gal model of a 3-day or 5-day reaction, and showed significant differences (*P* < 0.01 or *P* < 0.001) compared to the control. However, these compounds showed a minor effect after a 7-day incubation. It was also found that C or D could lower the CML formations in the BSA/glc model and showed significant differences (*P* < 0.05 or *P* < 0.01) compared to the control after a 3-day, 5-day and 7-day reaction. It was found that A, I, C, or D, each at 0.5 mM or 5 mM, could attenuate the AP formations in the BSA/MGO model of a 3-day reaction and showed significant differences (*P* < 0.001) compared to the control.

**Conclusions:**

The results suggest the potential anti-glycation activities of A and I *in vitro* may apply to cell models at higher glucose concentrations or to diabetic animal models, and need further investigation.

**Electronic supplementary material:**

The online version of this article (doi:10.1186/1999-3110-54-6) contains supplementary material, which is available to authorized users.

## Background

Acteoside (A) or its structural isomer of isoacteoside (I), a phenylethanoid glycoside containing caffeic acid (C), 3′,4′-dihydroxyphenylethanol (D), glucose, and rhammose, is reported in many plants and herbal medicines. Among these are *Ligstrum purpurascens* (Wong et al., [Bibr CR36][Bibr CR37]), *Brandisia hancei* (He et al. 2001), *Veronica persica* (Harput et al. 2002), *Pedicularis striata* (Chen et al. 2002), *Cistanche salsa* (Pu et al. 2003), *Buddleja officinalis* (Lee et al. 2005), *Callicarpa dichotoma* (Koo et al. 2005,2006;Lee et al. 2006b), *Clerodendron trichotomum* (Lee et al. [Bibr CR22][Bibr CR24]), *Cistanche deserticola* and *Boschniakia rossica* (Wu et al. 2006), *Scrophularia ningpoenis* (Huang et al. 2008), *Rehmannia glutinosa* (Li et al. 2006), and *Harpagophytum procumbens* (Boje et al. 2003). Acteoside was reported to exhibit antioxidant activities (Wong et al. 2001b) from bitter tea (*Ligstrum purpurascens*), a popular beverage in southern China. A and/or I were reported to have antioxidant, anti-hemolytic activities, angiotensin converting enzyme inhibitory activities, and anti-hypertensive activities using spontaneously hypertensive rats as models (Chen et al. 2012). Koo et al. (2006) reported that A and its aglycones effectively scavenge 1,1-diphenyl-2-picryl-hydrazyl and nitric oxide *in vitro*. It was reported that A and I inhibited IL-1β-activated expression of intercellular CAM-1 and vascular CAM-1 in human umbilical vein endothelial cells (Chen et al. 2009). Acteoside also showed protections against hydroxyl radical-induced oxidative stress of bovine pulmonary endothelial cells (Chiou et al. 2004) and inhibitions to nitric oxide and TNF-α productions throughg blocking AP-1 activations in lipopolysaccharide-stimulated macrophages (Lee et al. 2005;Rao et al. 2009).

Reactive oxygen species (ROS)–mediated reactions are closely associated with cardiovascular diseases, neurodegenerative diseases and other chronic diseases (Ames 1983;Gey 1990). In cells, respiratory chain reactions and glycation processes are important sources of ROS production. Glycation involves the nonenzymatic modification of proteins (especially lysine or arginine residues) through the reduction of sugars both *in vitro* and *in vivo,* and their metabolized intermediates, such as glyoxal or methylglyoxal (MGO). This induces the irreversible formation of advanced glycation end products (AGE), which may alter protein function and protein degradation. AGE can bind to the receptor for AGE (RAGE) on the membrane to elevate ROS productions through activation of NADPH oxidase (Calcutt et al. 2009). AGE-RAGE interaction may overgenerate intracellular ROS, which retards glucose uptake into adipocytes and then induces monocyte inflammatory factor, chemoattractant protein-1, to participate in the development of obesity-related insulin resistance (Unoki and Yamagishi 2008). Wu et al. (2011) reported that the MGO-mediated glycated fetal bovine serum can increase intracellular ROS and IL-1β expressions in THP-1 monocytes, and TNF-α expressions in RAW264.7 macrophages. They proposed that MGO-mediated glycated fetal bovine serum can cause oxidative and inflammatory injury of monocytes, macrophages and vascular endothelial cells. Several AGEs have been identified, including *N*^ϵ^-(carboxymethyl)lysine (CML), *N*^ϵ^-(carboxyethyl)lysine, pyrraline, pentosidine, crossline, imidazolones, glyoxal-lysine dimer, methylglyoxal-lysine dimer, Arg-Lys imidazole, argpyrimidine (AP), etc. (Lapolla et al. 2006). It is suggested that AGE formations and accumulations are correlated with cardiovascular disease, insulin resistance and diabetic complications (Unoki and Yamagishi 2008). In this study, A, I, and their constituents, C and D, were used to investigate the inhibitory activity against AGE formations *in vitro* detected by anti-CML, using bovine serum albumin (BSA)/glucose (glc) and BSA/galactose (gal) as models or anti-AP using BSA/MGO as models. BSA/glc and BSA/gal were recognized as early stages of AGE formations; BSA/MGO were recognized as middle stages of AGE formations (Wu and Yen 2005). It was found that the potential anti-glycation activities of A and I *in vitro* may apply to cell models at higher glucose concentrations or to diabetic animal models and needed further investigation.

## Methods

### Materials

Natural compounds of A and I were purchased from Xinma Bio-Tech Co., LTD (purities > 98%, Shanghai, China). The 3′,4′-dihydroxyphenylethanol was obtained from Tokyo Chemical Industry Co., LTD (Tokyo, Japan). Bovine serum albumin (BSA, 2 mg/ml, No. 23209) was obtained from Thermo Fisher Scientific Inc. (Rockford, IL). Gal, Glc, and caffeic acid were purchased from Sigma Chemical Co. (St. Louis, MO). Anti-*N*^ϵ^-(carboxymethyl)lysine (anti-CML) polyclonal antibody (ab27684) was obtained from Abcam Inc. (Cambridge, MA), and anti-argpyrimidine (anti-AP) monoclonal antibody was purchased from Cosmo Bio Co. Ltd. (Tokyo, Japan), and horseradish peroxidase (HRP)-conjugated goat anti-mouse IgG and HRP-conjugated goat anti-rabbit IgG were from Sigma Chemical Co. (St. Louis, MO, USA).

### BSA/glc and BSA/gal *in vitro* as anti-protein glycation screening models

The BSA/glc or BSA/gal models for protein glycation experiments *in vitro* (Ahmad et al. 2007;Ledesma-Osuna et al. 2008) were followed with some modifications. The total 100 μl of reaction solution contained 20 μl of BSA solution (2 mg/ml), 60 μl of 1M gal or glc solution, 10 μl of PBS (10-fold dilutions in the final), and 10 μl of DMSO or tested compounds (A, I, C and D, each 50 mM, and 5 mM in the final). The blank test contained BSA only, and the control test contained BSA/gal or BSA/glc under the same conditions. These mixtures were placed in a 37°C water bath for the desired number of days (3, 6, 10, or 14 days for glycation experiments or 3, 5, or 7 days for anti-glycation experiments with tested compound additions), and then were stored at 4°C for further use. After that, a 24 μl of each reaction solution was mixed with 6 μl of sample buffer (5-fold dilution) and heated at 100°C for 5 min. An aliquot of 15 μl was then added to each well of 10% sodium dodecyl sulfate-polyacrylamide for gel electrophoresis. After electrophoresis, the gels were cut into 2 parts. One part was fixed with 12.5% trichloroacetic acid for 30 min and then stained with Coomassie brilliant blue G-250; the other part was equilibrated in Tris-glycine buffer (pH 8.3) and transferred onto immobile polyvinylidene difluoride (PVDF) membranes (Millipore, Bedford, MA, USA) for immunostaining. The PVDF membranes were blocked with 1% gelatin in NaCl/EDTA/Tris (NET) solution for 1 h at room temperature and incubated overnight at 4°C. An anti-CML antibody was used at a 5000-fold dilution. The PVDF membrane was washed 3 times for 10 min with PBS containing Tween-20 (PBST). Next, HRP-conjugated secondary antibody solution (5000-fold dilution in 0.25% gelatin in NET solution) was added, and the membrane was washed with 1× PBST. Immunoblots were stained by using the HRP-hydrogen peroxide-aminoethyl carbazole system. For quantifying CML formations in the BSA/glc or BSA/gal models, an enzyme-linked immunosorbent assay (ELISA) was performed. A 100-μl aliquot containing 5-μl of the reaction solution or 100-μl of 10-fold diluted PBS was loaded into a high-binding 96-well microtiter plate (Nunc MaxiSorp, type F, Roskilde, Denmark); the plate was covered with an adhesive strip and incubated at 37°C for 2 h, washed thrice for 10 min each with 200 μl of PBST, and then blocked with 100 μl of NET solution at 37°C for 30 min. A 100-μl anti-CML polyclonal antibody solution (5000-fold dilution in 0.25% gelatin in NET solution) was added and the samples were incubated at 4°C overnight, followed by washing of the plates thrice for 10 min each with 200 μl of PBST. Next, 100 μl of HRP-conjugated goat anti-rabbit IgG solution (5000-fold dilution in 0.25% gelatin in NET solution) was added, and the samples were incubated at 37°C for 1 h; subsequently, the plate was washed again using 200 μl of PBST. A staining solution was prepared by dissolving 1 mg of 3,3′,5,5′-tetramethylbenzidine in 1 ml of DMSO, followed by addition of 5 μl of 30% hydrogen peroxide; the volume was increased to 10 ml with 50 mM PB, pH 5.0. A 100-μl aliquot of staining solution was added to the plate; this reaction was allowed to proceed in the dark at 37°C for 30 min. The reaction was stopped by adding 25 μl of 1 M HCl, and the absorbance at 450 nm was measured by using an ELISA reader (TECAN Sunrise microplate reader; Männedorf, Switzerland). For anti-protein glycation in the presence of A, I, C, or D, the relative CML formed index (%) was expressed and the CML formation in the control was recognized as 100%.

### BSA/MGO as models for anti-protein glycation *in vitro*

(Wu and Yen The BSA/MGO models for protein glycation experiments *in vitro*^2005^;Wu et al. 2011) were followed with some modifications. The total 100-μl reaction solution contained 20 μl of BSA solution (2 mg/ml), 10 μl of PBS (10-fold dilutions in the final), 10 μl of 10 mM or 50 mM MGO, 50 μl of tested compounds (A, I, C and D, each 1 mM or 10 mM), and distilled water. The blank test contained BSA only, and the control test contained BSA/MGO under the same conditions. These mixtures were placed in a 37°C water bath for the desired number of days (2 or 3 days for glycation experiments or 3 days for anti-glycation experiments with tested compound additions), and then stored at 4°C for further use. After that, an 8 μl of each reaction solution was mixed with 2 μl of sample buffer (5-fold dilution) and was then heated at 100°C for 5 min; an aliquot of 5 μl was added to each well of 10% sodium dodecyl sulfate-polyacrylamide gel in preparation for electrophoresis. After electrophoresis, the gels were cut into 2 parts; one was fixed with 12.5% trichloroacetic acid for 30 min and then stained with Coomassie brilliant blue G-250, while the other part was equilibrated in Tris-glycine buffer (pH 8.3) and transferred onto immobile PVDF membranes (Millipore, Bedford, MA, USA) for immunostaining. An anti-AP monoclonal antibody and HRP-conjugated secondary antibody solution were used at a 5000-fold dilution. Immunoblots were detected using Western Chemiluminescent HRP Substrate kits containing luminol reagents and peroxide solutions (no. WBKL S0050; Immobilon™, Millipore). Each blot was imaged using the Syngene GeneGnome5 imaging system (Syngene, Cambridge, UK) equipped with the GeneSys/GeneTools software (Syngene). For quantifying AP formations in the BSA/MGO models, an ELISA was performed. A 100-μl aliquot containing 2-μl of the reaction solution or 100-μl of 10-fold diluted PBS was loaded into a high-binding 96-well microtiter plate (Nunc MaxiSorp, type F, Roskilde, Denmark). The 100-μl anti-AP monoclonal antibody solution (5000-fold dilution in 0.25% gelatin in NET solution) and 100 μl of HRP-conjugated goat anti-mouse IgG solution (5000-fold dilution in 0.25% gelatin in NET solution) were used for detections. The ELISA staining procedure was the same as above-mentioned, and the absorbance at 450 nm was measured by using an ELISA reader (TECAN Sunrise microplate reader; Männedorf, Switzerland). For anti-protein glycation in the presence of A, I, C, or D, the relative AP formed index (%) was expressed and the AP formation in the control (BSA/MGO) was recognized as 100%.

### Statistical analyses

The mean ± SD values were calculated from triplicate measurements. The difference between the control and the experimental group at the same treated time or the saqme concentration was analyzed using Student’s *t*-test, and the *P*-value of less than 0.05 (*), 0.01 (**), and 0.001 (***) were recognized as different significantly. The statistical analysis was performed using the GraphPad Prism Software 5.0.

## Results and discussion

**Figure 1:**
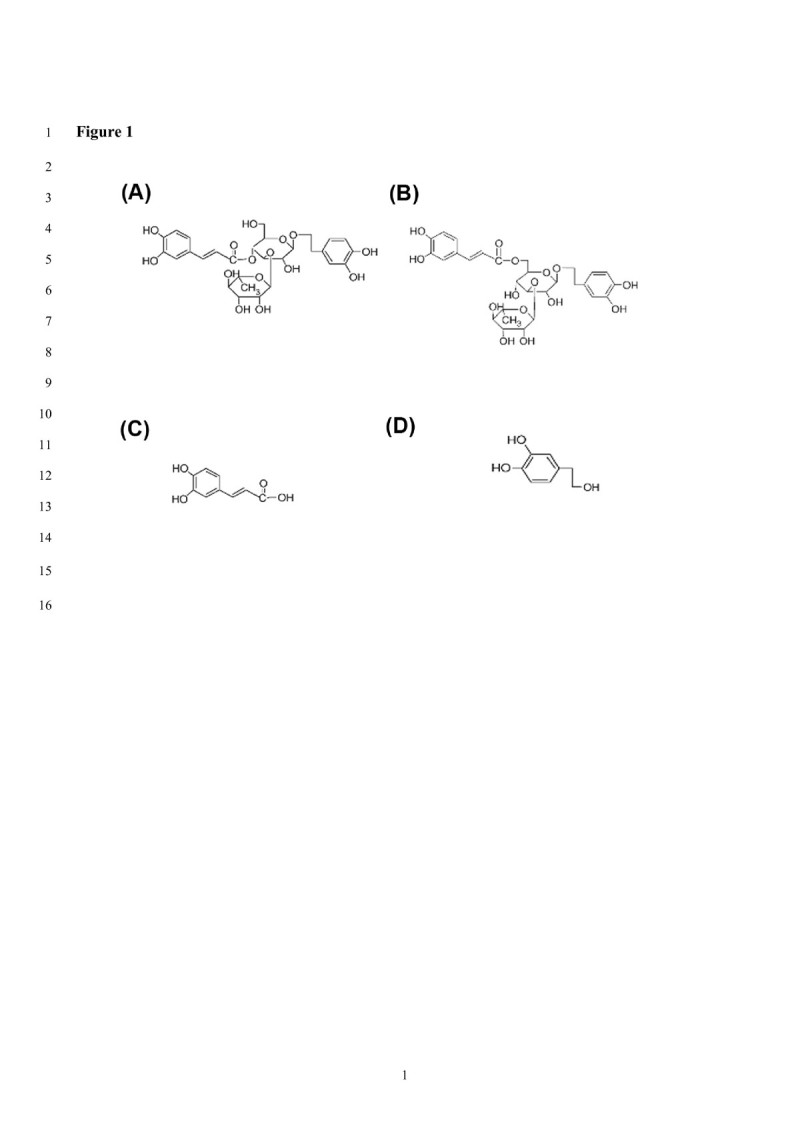
**The structures of (A) acteoside, (B) isoacteoside, (C) caffeic acid, and (D) 3′,4′-dihydroxyphenylethanol used in the experiments.**

A and I were reported to have antioxidant activities *in vitro*, and A also exhibited antihypertensive activities in spontaneously hypertensive rat models (Chen et al. 2012). In this study, A, I, and the constituents C and D, were used to investigate the inhibitory activity against AGEs formations *in vitro* detected by anti-CML using BSA/glc and BSA/gal as models or by anti-AP using BSA/MGO as models. Figure 1 shows the structure of A, I, C, and D.

**Figure 2:**
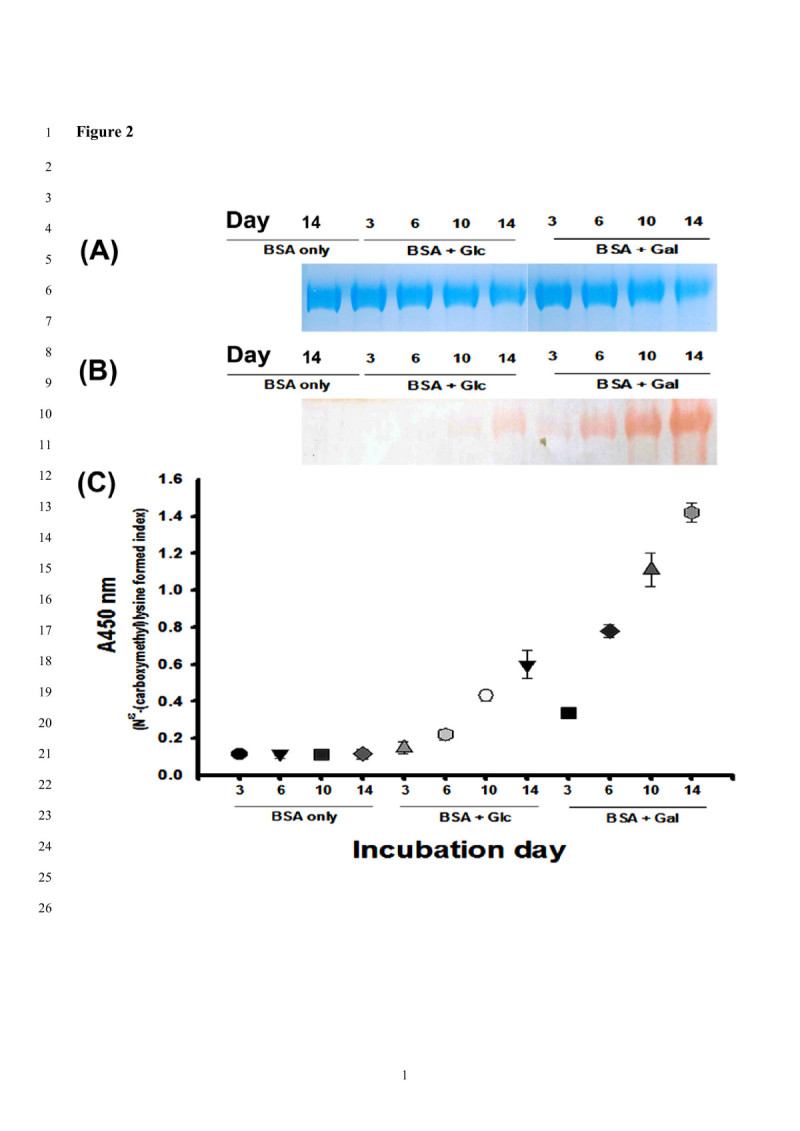
**The bovine serum albumin (BSA) glycation formations were detected at different days (3, 6, 10, and 14 days) in BSA/glucose (glc) or BSA/galactose (gal) models. (A)** The protein stains in SDS-PAGE gels by Coomassie brilliant blue G-250; **(B)** The immune stains by anti-*N*^ϵ^-(carboxymethyl)lysine (anti-CML) antibody; **(C)** The ELISA method for CML formations detected by anti-*N*^ϵ^-(carboxymethyl)lysine antibody.

The CML was reported to be the most thoroughly studied with respect to the chemical and biological properties of AGE (Nagai et al. 2013). In the beginning, the protein glycation formations were tested at different days (at 3, 6, 10 and 14 days) in the BSA/glc or BSA/gal models. Figure 2(A) shows the protein stains; Figure 2(B) shows the immune stains. Compared to the BSA only, at day 14 of incubation (the left lane), the protein band of BSA (Figure 2(A)) was gradually faded after prolonged incubation (at the 10- or 14-day reaction point), while the CML formations in the same BSA protein band position gradually increased (Figure 2(B)) as detected by Western blotting using the anti-CML antibody. The CML formations were accelerated in the BSA/gal models more than in the BSA/glc models, and the CML formations were clearly stained after the 6-day incubation period in the BSA/gal models. However, the 14-day incubation in the BSA/glc models was required for detection of CML formations. On the other hand, the versatile ELISA method was developed for protein glycation formations detected by the anti-CML antibody in the BSA/glc or BSA/gal models as showed in Figure 2(C). Compared to the BSA only, at 3-, 6-, 10- and 14-day incubations (as the blank), the CML formations in BSA/glc or BSA/gal increased as the days of incubation increased. The CML formations could be detected by the ELISA method in the BSA/glc models only at 3-day incubation. The number of CML formations (CML formed index) in the BSA/glc models after 14-day incubations were between those of the 3-day and 6-day incubations of the BSA/gal models. The CML formations in the ELISA data (Figure 2(C)) were comparable to those of the immune stains (Figure 2(B)) in Western blotting. The _D_-glc or _D_-gal was reported to react with proteins/peptides both *in vitro* and *in vivo*, as well as with their metabolized intermediates such as glyoxal or MGO, leading to AGE formations through nonenzymatic glycation (Song et al. 1999;Parameshwaran et al. 2010;Tsai et al. 2011). The reaction rate of _D_-gal is approximately 4.7-fold of the reactivity of _D_-glc toward hemoglobin *in vitro* (Burn and Higgins 1981). Therefore, A, I, C and D were used to evaluate the effects on anti-protein glycation.

**Figure 3:**
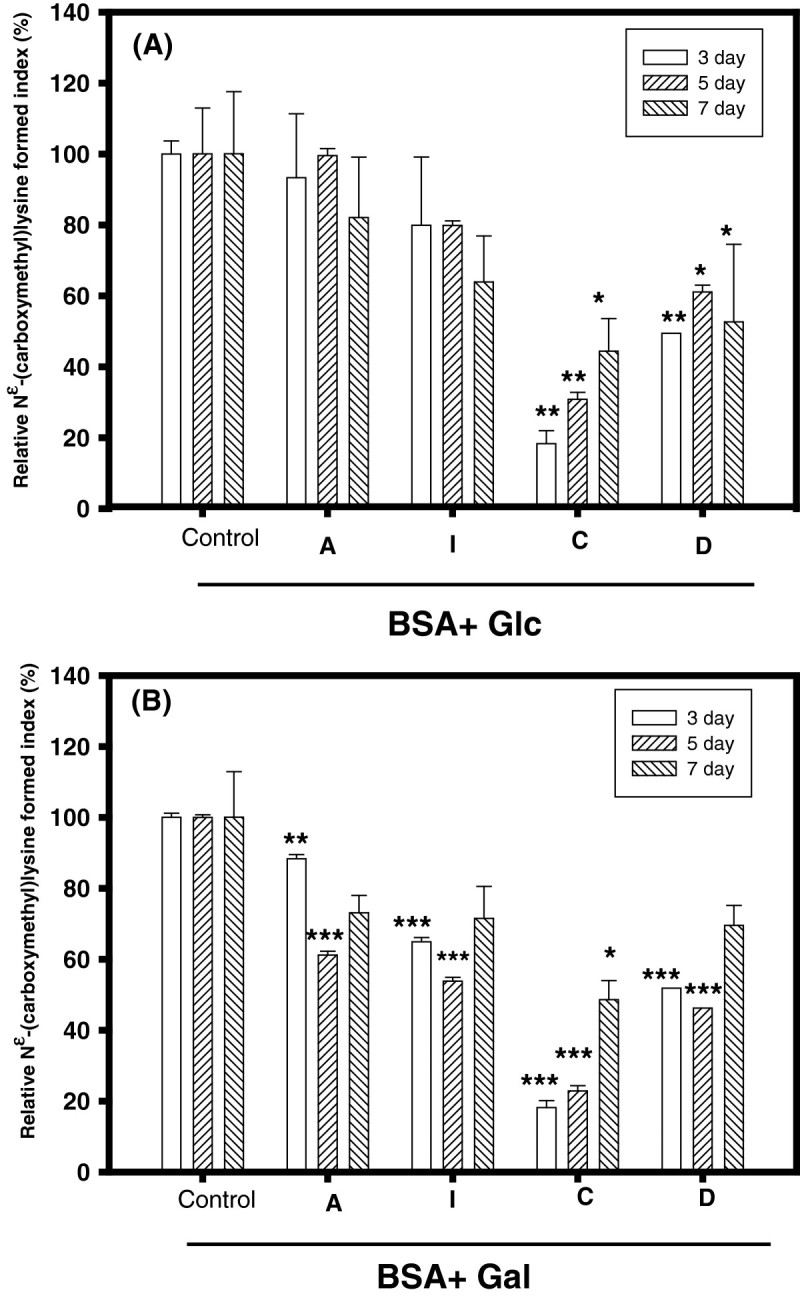
**Effects of acteoside (A), isoacteoside (I), caffeic acid (C), and 3′,4′-dihydroxyphenylethanol (D) on anti-CML formatiopns in different incubation days (3, 5, and 7 days) at (A) BSA/glc modes or (B) BSA/gal models. Each tested sample was 5 mM in the final concentration.** The mean ± SD values were calculated from triplicate measurements. The difference between the control and the experimental group at the same treated time was analyzed using Student’s *t*-test, and the *P*-value of less than 0.05 (*), 0.01 (**), and 0.001 (***) were recognized as different significantly.

Figure 3 shows the anti-CML formations of A, I, C and D each being at 5 mM in the BSA/glc models (Figure 3(A)) and the BSA/gal models (Figure 3(B)) after 3-day, 5-day, and 7-day incubations by the ELISA method. In the BSA/glc models, it was found that A or I additions showed no significant difference (*P* > 0.05) in CML formations compared to the control. However, the constituent of C or D showed a significant difference (*P* < 0.05 or *P* < 0.01) in CML formations compared to the control after 3-day, 5-day and 7-day incubations (Figure 3(A)). In the BSA/gal models, A, I, C and D additions showed significant differences (*P* < 0.01 or *P* < 0.001) in CML formations compared to the control after 3-day and 5-day incubations, but not after 7-day incubations (Figure 3(B)). This meant that the anti-protein glycation activity of A or I was originally from C or D. Fujiwara et al. (2008) reported that natural compounds, including: acteoside, quercetin, and quercetin 3-sambubioside, could stimulate CML formations, and sophoradiol and lupeol could lower CML formations detected by the anti-CML antibody using the ELISA method in BSA (2 mg/ml) and ribose (33 mM) in PBS at 37°C for 7-day incubations. Our present results of A or I with anti-CML formations at BSA/gal models differed from those reported by Fujiwara et al. (2008); the different glycation systems (BSA/gal vs. BSA/ribose) and different incubation periods (3-day or 5-day vs. 7-day) might be reasons for these differences. Wu and Yen (2005) reported that flavonoids, including: luteolin, quercetin, rutin, etc., could lower AGE formations detected by glycated protein fluorescence (Ex 330 nm and Em 410 nm) in BSA (50 mg/ml) and glc (0.8 M) in 1.5 M PB (pH 7.4) at 37°C for 7-day incubations, and they proposed that the hydroxyl group at the C-3′ position of flavonoid and/or antioxidant properties contributed to the inhibitory activities against AGE formations. The A or I and its constituents of C and D had one hydroxyl group in catechol (3,4-dihydroxyphenol) moiety, and the A and I were reported to have antioxidant activities (Chen et al. 2012). This was the same as the proposal of Wu and Yen (2005) for anti-protein glycation. Nagai et al. (2013) reviewed and proposed that a molecule with the catechol group (such as gallic acid, quercetin, isoquercetin, luteolin 7-O-glucoside and acteoside) under high concentrations (such as 1 mM) may enhance CML formations, and low concentrations (such as 0.01 mM) may attenuate CML formations. However, these proposals were not followed by a series of structure-activity relationship investigations.

**Figure 4:**
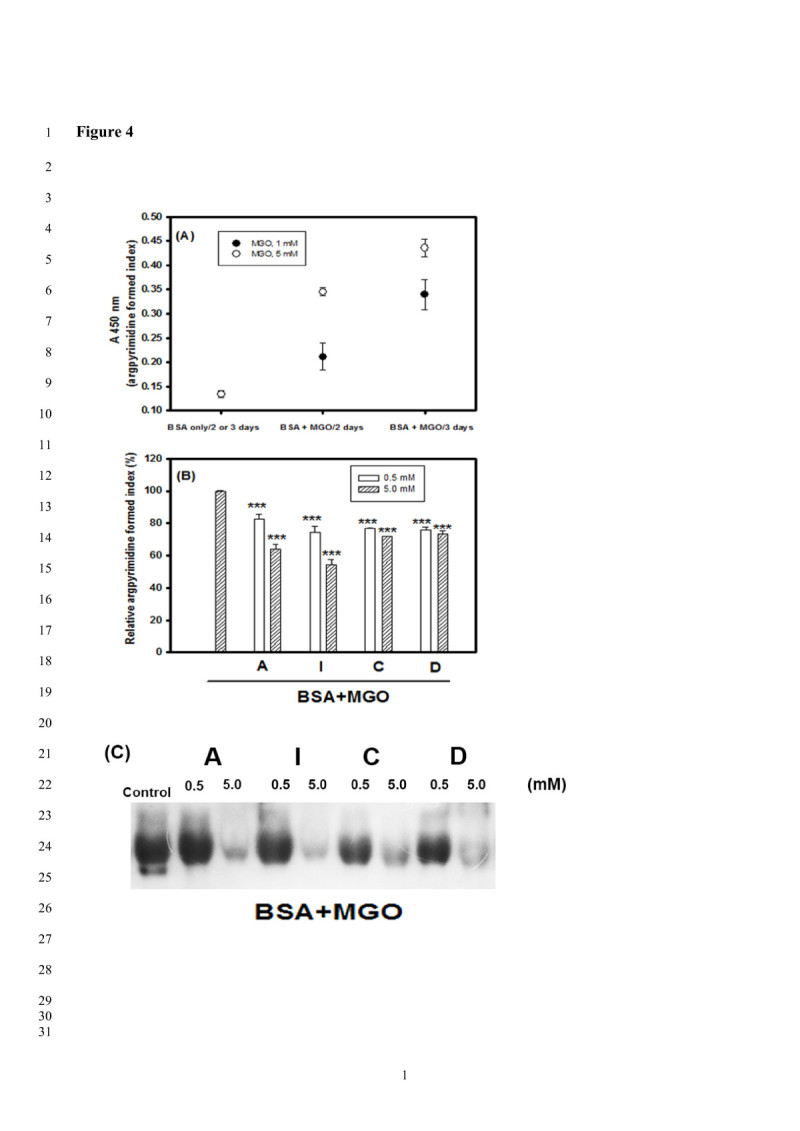
**Effects of acteoside (A), isoacteoside (I), caffeic acid (C), and 3′,4′-dihydroxyphenylethanol (D) on anti-argpyrimidine (anti-AP) formatiopns in different incubation days (2 or 3 days) at BSA/methylglyoxal (MGO) models. (A)** The AP formations in BSA under different MGO concentrations (1 or 5 mM in the final concentrations) for 2 or 3 days using ELISA methods detected by anti-argpyrimidine (anti-AP) antibody; **(B)** Effects of 0.5 or 5 mM concentrations of A, I, C, and D on anti-AP formations for 3-day in BSA/MGO (5 mM) models detected by ELISA methods; **(C)** The immune stains on anti-AP formations for 3-day in BSA/MGO (5 mM) models detected by anti-AP antibody. The mean ± SD values were calculated from triplicate measurements. The difference between the control and the experimental group at the same concentration was analyzed using Student’s *t*-test, and the *P*-value of less than 0.05 (*), 0.01 (**), and 0.001 (***) were recognized as different significantly.

Figure 4 shows the anti-AP formations of A, I, C and D in the BSA/MGO models. Padayatti et al. (2001) reported that MGO could rapidly interact with Arg protein residues, but not with Lys protein residues, to generate AP formations, which were at high levels in brunescent cataractous lenses, a diabetic complication. In the beginning, the AP formations were detected by the ELISA method in different MGO concentrations (1 or 5 mM) for 2- or 3-day incubations (Figure 4(A)). It was found that higher MGO concentrations may increase higher levels of AP productions (AP formed index A450 nm) in BSA/MGO models under the same incubation days. Therefore, BSA/5 mM MGO for 3-day incubations were selected for further investigation. It was found that A, I, C and D at 0.5 mM or 5 mM could lower AP formations (Figure 4(B)), and showed significant differences (*P* < 0.001) compared to the control (BSA/5 mM MGO for 3-day incubations) using the ELISA method and detection by the anti-AP antibody. The use of Western blot revealed the BSA glycations (BSA/5 mM MGO for 3-day incubations) detected by AP antibodies. It was found that the A, I, C and D at 5 mM showed clear reductions of AP formations in BSA positions, and C and D at 0.5 mM also showed anti-AP formation activities (Figure 4(C)). The proanthocyanidins from cinnamon bark were reported to have scavenging activities against MGO to form MGO-procyanidin adducts and then prevented AGE formations (Peng et al. 2008).

From the results shown in Figures 3 and 4, we proposed that the anti-protein glycation activities of A and I may result from the constituents of C or D. It was reported that C exhibited antioxidant activity (Chen and Ho 1997) and anti-glycation activity against BSA/MGO models (Gugliucci et al. 2009). The constituent D was reported to have protective roles against hydrogen peroxide–induced cell cytotoxicity (Manna et al. 1997). The antioxidant activities of C and D may explain in part the anti-protein glycation activities. Wu et al. (2011) reported that the constituent C exhibited proglycation effects (or pro-oxidant activity detected by electron spin resonances), which were enhanced by metal ions (such as Fe^2+^ and Cu^2+^) and abolished by EDTA, reduced glutathione, or α-lipoic acid in BSA/MGO models. The present results were similar to those of Gugliucci et al. (2009) and different from those of Wu et al. (2011). This might be due to the detection method used (antibody detection vs. protein cross-linking in SDS-PGAE gels) and the number of incubation days (3-day vs. 7-day). The uses of antibody to detect the AGE formations in the present results are more specific than in other reported results detected by glycated protein fluorescence (Ex 330 nm and Em 410 nm). However, the immune stain used in the present research cannot cover all forms of chemically identified AGE. It is noted that the anti-glycation activity does not show a positive correlation with the diabetic treatments (Nagai et al. 2013). Therefore, it is suggested that the cell experiments and diabetic animal models should be performed to clarify the effects of the natural products of A, I, C and D on anti-diabetic complications and/or anti-diabetes treatment.

## Conclusions

In conclusion, A and I together with its consittuents, C and D, exhibited anti-protein glycation activities in different model systems *in vitro*. The results suggest the potential anti-glycation activities of A and I *in vitro* may apply to cell models in higher glucose concentrations or diabetic animal models and need further investigations.

## Authors’ original submitted files for images

Below are the links to the authors’ original submitted files for images. Authors’ original file for figure 1 Authors’ original file for figure 2 Authors’ original file for figure 3 Authors’ original file for figure 4

## Abbreviations

A: Acteoside
AGEs: Advanced glycation endproducts
AP: Argpyrimidine
BSA: Bovine serum albumin
C: Cafeic acid
CML: N^ϵ^-(carboxymethyl)lysine
D: 3′,4′-dihydroxyphenylethanol
ELISA: Enzyme-linked immunosorbent assay
Gal: Galactose
Glc: Glucose
HRP: Horseradish peroxidase
I: Isoacteoside
MGO: Methylglyoxal
NET: NaCl/EDTA/Tris
PVDF: Polyvinylidene difluoride
ROS: Reactive oxygen species.
