# Supplementary figures and images for: Inhibitory activities of acteoside, isoacteoside, and its structural constituents against protein glycation in vitro
Source: Bot Stud. 2013 Aug 19;54:6. doi: 10.1186/1999-3110-54-6 (PMC5432847; doi:10.1186/1999-3110-54-6)

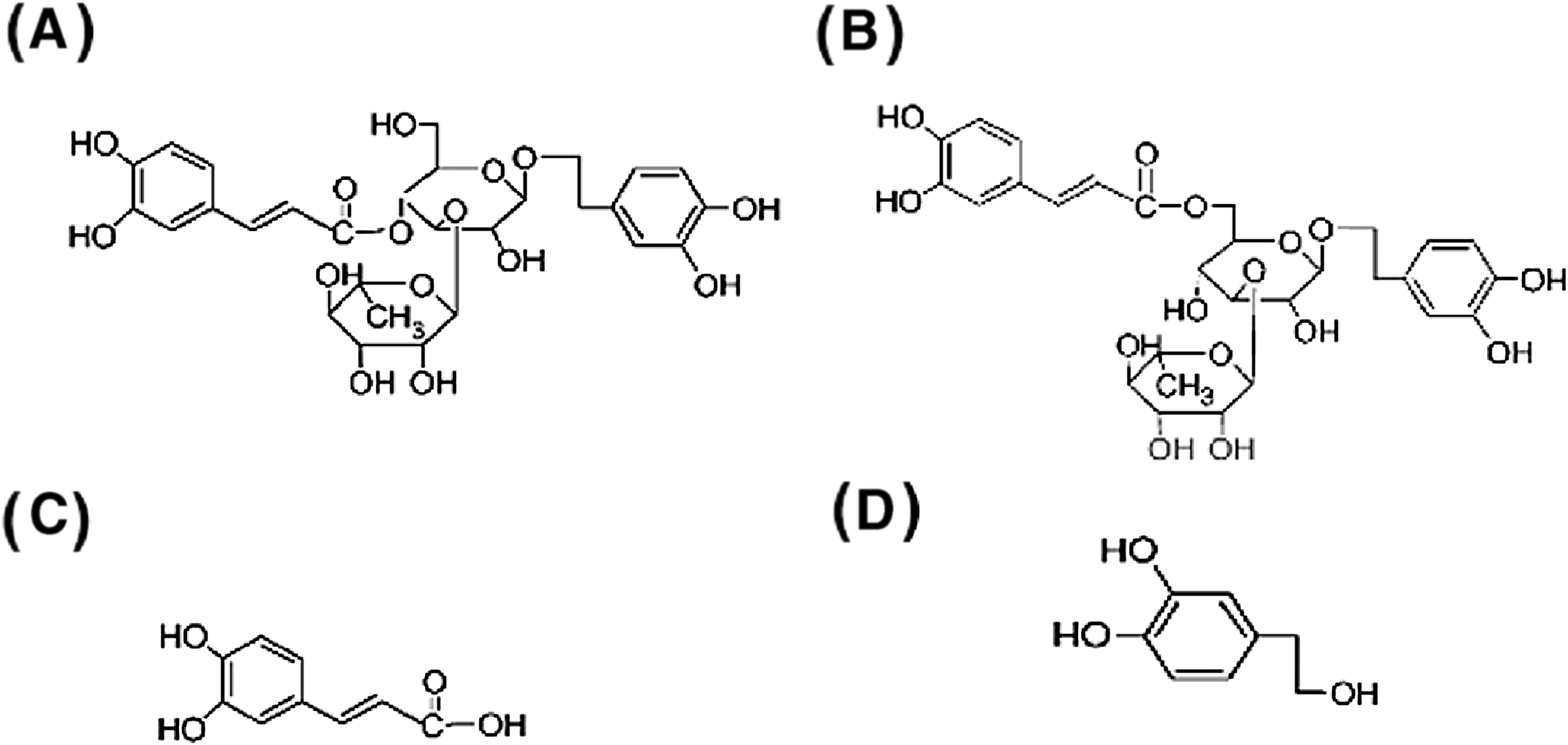

Supplement: Supplementary file 1 — Authors’ original file for figure 1 [file 40529_2013_6_MOESM1_ESM.tif]

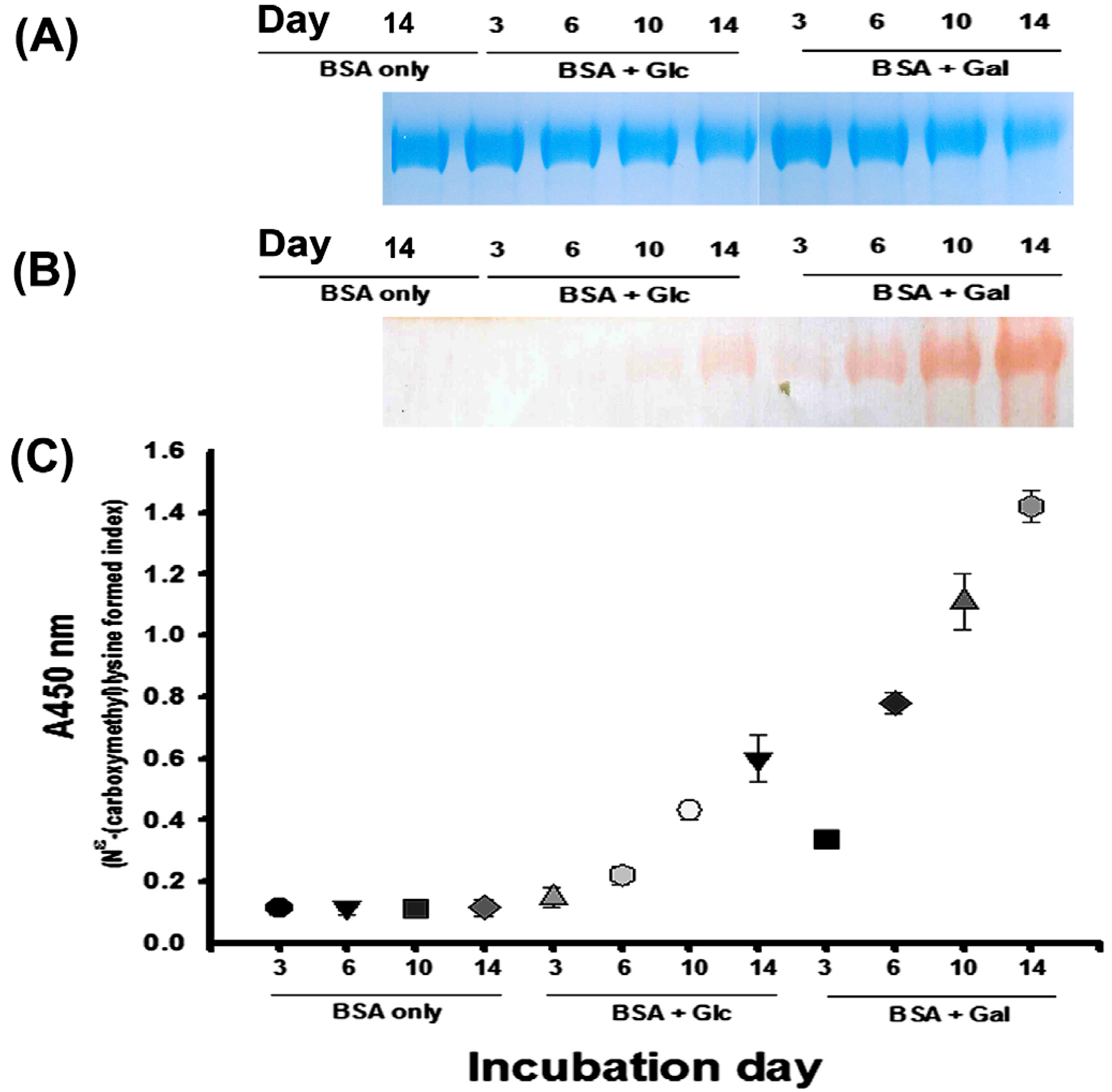

Supplement: Supplementary file 2 — Authors’ original file for figure 2 [file 40529_2013_6_MOESM2_ESM.tif]

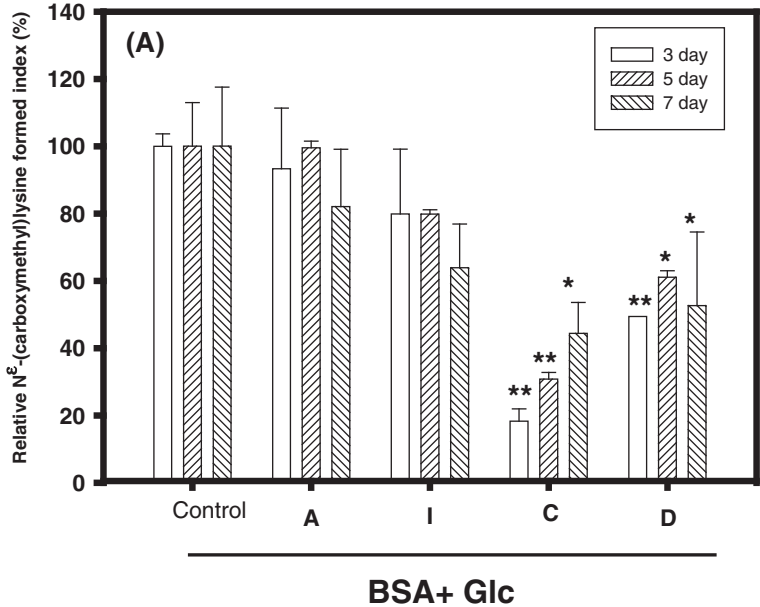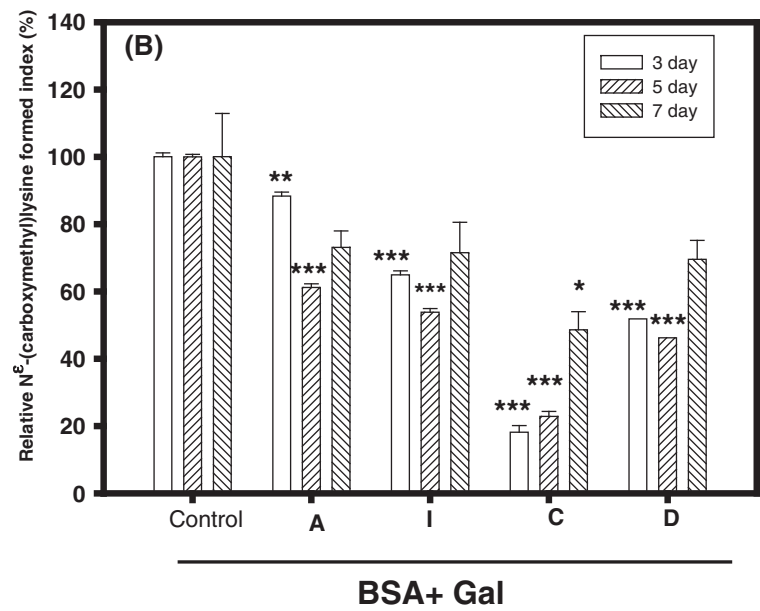

Supplement: Supplementary file 3 — Authors’ original file for figure 3 [file 40529_2013_6_MOESM3_ESM.pdf]

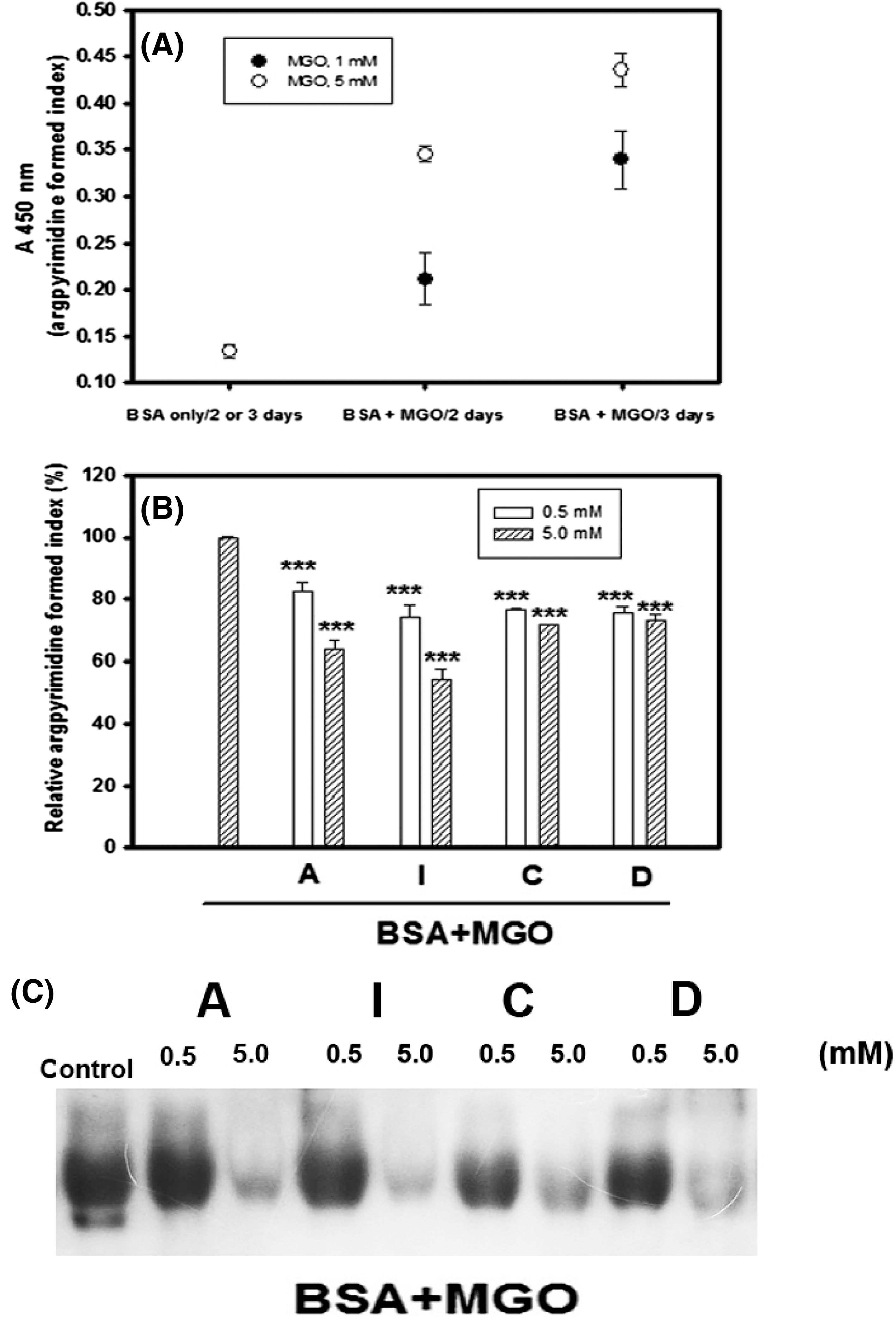

Supplement: Supplementary file 4 — Authors’ original file for figure 4 [file 40529_2013_6_MOESM4_ESM.tif]
